# Using deep-learning algorithms to derive basic characteristics of social media users: The Brexit campaign as a case study

**DOI:** 10.1371/journal.pone.0211013

**Published:** 2019-01-25

**Authors:** Moreno Mancosu, Giuliano Bobba

**Affiliations:** 1 Collegio Carlo Alberto, Turin, Italy; 2 Department of Cultures, Politics and Society, University of Turin, Turin, Italy; Centre National de la Recherche Scientifique, FRANCE

## Abstract

A recurrent criticism concerning the use of online social media data in political science research is the lack of demographic information about social media users. By employing a face-recognition algorithm to the profile pictures of Facebook users, the paper derives two fundamental demographic characteristics (age and gender) of a sample of Facebook users who interacted with the most relevant British parties in the two weeks before the Brexit referendum of 23 June 2016. The article achieves the goals of (i) testing the precision of the algorithm, (ii) testing its validity, (iii) inferring new evidence on digital mobilisation, and (iv) tracing the path for future developments and application of the algorithm. The findings show that the algorithm is reliable and that it can be fruitfully used in political and social sciences both to confirm the validity of survey data and to obtain information from populations that are generally unavailable within traditional surveys.

## Introduction

Political science research using social media online data has constantly grown in recent years. These studies have mainly addressed the questions of predicting elections [1–3], assessing the sentiments of different audiences [4], studying online deliberations [5, 6], and understanding the relationship between the use of social media and type of political participation and online behaviour [7–9].

To date, many of the studies on politics and social media have focused on Twitter, primarily because of the public nature of the tweets posted on the platform. In addition to studies on Twitter’s communicative features, a large number of scholars have focused on the relevance of this social media for forecasting public opinion trends and political behaviour [10–12]. These studies rely on the implicit assumption that Twitter users are demographically representative of the voting-age population, or at least that Twitter conversations represent the opinions of the electorate. Facebook has also been the subject of numerous studies addressing users’ political participation and political preferences. Due to the restrictive privacy policy of the platform, scholars have carried out their research mainly through surveys and experimental methods [13–18].

A recurrent criticism concerning the use of online social media data in political science research, however, is the lack of demographic information about social media users. Indeed, social media data are only able to assess what users do, and not who they are (in terms of gender, age, educational level, social class, etc.). Social media data are thus evaluated as ‘incomplete’, because they do not provide an exhaustive amount of information that is crucial for social and political research (information, for instance, that is almost always provided by surveys). To solve this endemic drawback in this part of the research, many attempts have been made to derive relevant pieces of information (such as gender, age, occupation, etc.) from social media users. We can list at least three approaches to the issue: a first method is based on meta-data, namely, the data that are made public by users themselves and are downloadable by employing social networks’ APIs—and, more importantly, without violating the terms and conditions that regulate user behaviour on social media. Different studies, for example, used information that is (optionally) self-reported and made visible by Twitter users (i.e. name and location) in the US to develop techniques to infer the geographic distribution, gender and race/ethnicity of the Twitter population [19]. They found that Twitter users significantly “overrepresent the densely population regions of the U.S., are predominantly male, and represent a highly non-random sample of the overall race/ethnicity distribution” [19]. In comparison, by relying on the short bio that every Twitter user is invited to write, others [20] inferred the age, gender and social class of a rather small (less than 1%) portion of the Twitter sphere.

A second approach, much more demanding in terms of computational power and the amount of data available for each user, aims at deriving key traits of individuals by analysing their vocabulary. The best illustration of this type of study is represented by the works of the COSMOS (Collaborative Social Media Observatory: www.cosmosproject.net) scholars. Schwartz and colleagues [21]—but see also [22]—developed a series of techniques to derive information on gender, age and mood of social network users, after having collected a relevant amount of their statuses. Although the findings are very promising, the very substantial amount of data needed makes this type of research very challenging. Also, it is extremely difficult to apply these techniques to the vast majority of situations in the political analysis of social media, especially when dealing with small bits of data (such as ‘likes’, reactions, comments, etc.).

A third approach aims at retrieving users’ information by asking them directly through surveys collected among samples of Facebook users [23, 24]. The construction of the sample is the crucial issue. However, since no comprehensive list of Twitter users or their socio-demographic characteristics is publicly available, how could a representative sample be constructed? Vaccari and colleagues [9] tried to solve this drawback by studying Twitter users who tweeted messages regarding the German and Italian elections of 2013. Once detected through a keyword search, a randomly selected sample of users was invited to complete a survey; the final sample obtained amounted to 1,143 individuals in Germany and 1,493 in Italy (with a response rate of 4%). Although the sample could be considered representative of the specific population of people who posted at least one election-related tweet, it does not allow generalisation to other populations, such as Twitter users as a whole, or the electorate.

As it is possible to infer from this review, the vast majority of these approaches are applied on Twitter, while little research has been performed on other platforms, such as Facebook or Instagram [21], a social network that is much more important in terms of population coverage.

In this paper, to derive personal characteristics on social media, we propose to employ a piece of information usually neglected by the previous literature, but usually freely accessible on the most relevant social networks: the so-called ‘profile picture’ of the user, namely the picture by means of which the user present him/herself to the social media community (the picture is the first to be presented when a user comments, likes or shares a public post). Human faces are able to reveal a considerable amount of information about individuals, such as their mood, gender or age [25, 26]. Our main aim is to assess whether the faces that social media users voluntarily share allow us to obtain reliable information about them. To infer this information, we employ an automatised procedure, based on machine-learning algorithms—namely, a Convolutional Neural Network (CNN) algorithm. To test the heuristic validity of this technique, we apply it to a 2016 Facebook dataset containing social media data collected during the campaign for the United Kingdom European Union membership referendum (i.e. the Brexit referendum), held on 23 June 2016. We apply the algorithm to those people who, at least once, ‘liked’ the status of one of the main leaders/parties (Conservatives, Labour, and UKIP) during the last two weeks of the Brexit campaign. The article aims at (i) testing the precision of the algorithm, (ii) testing its validity, by assessing the consistency of CNN data with survey data, (iii) inferring new evidence on digital mobilisation, and (iv) tracing the path for future and further developments and application of the CNN algorithm.

First, for a subsample of profiles, we assess the validity of the measure obtained, by manually verifying whether the algorithm plausibly identifies the age and gender of the users. Then, by using BES 2016 panel data, we compare average age and gender distribution for “leavers” and “remainers” obtained with the algorithm with survey data—the “gold standard” in political research. As an example of the possible uses of this information, we analyse a topic previously investigated in the literature, concerning statuses that politicians usually employ to mobilise their supporters (also known as a ‘call to action’). Those stimuli tend to encourage users to like, share, sign petitions, or talk with friends about the issue raised by the party/leader’s status: although this kind of message has been demonstrated as generating greater involvement among the social media audience, and, thus, capable of distributing more political messages [27, 28], virtually no research has investigated even the basic characteristics of who is mainly activated by this kind of status: we show that, consistent with cohort explanations of political activism offline, older people are more likely than younger users to react to mobilising Facebook statuses. For what concerns the originality of the paper and its relevance, this contribution might be interesting for social scientists (sociologists, political scientists, communication scientists), for, at least, for two orders of reasons: first of all, the paper proposes, to our knowledge for the first time, a relatively accessible method to obtaining crucial information that would be impossible to obtain otherwise. This method seems also able to collect a larger amount of non-missing information compared to other methods (at least from what concerns political activity on social media). The second order of reason for which the paper might be interesting for a social science audience regards its substantive contribution. We explore a topic poorly developed in social media research, such as the gender/age gap in the responsiveness toward call-to-action claims, producing new results directly from the actual social media environment.

## Face-recognition algorithms

Over the past few years, face-recognition estimation has become an important topic in the computer vision industry and scientific debate, mainly because of the multiplicity of applications that these techniques have [26, 29–33]. The main assumption behind this family of techniques is that faces are able to indirectly convey numerous insights into an individual (such as his/her biological characteristics, mood, etc.).

**Fig 1:**
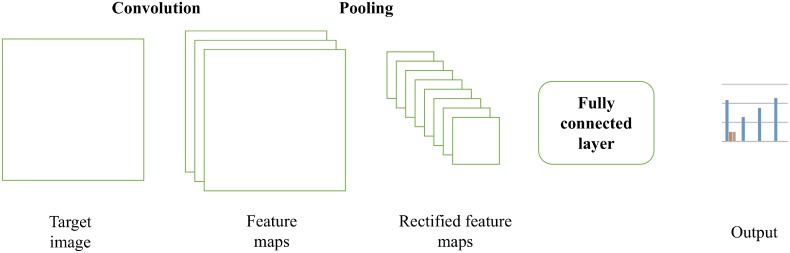
CNN algorithm for image recognition. An intuitive description.

Face-recognition and, more generally, image-recognition algorithms mostly rely on what is usually called machine-learning [34, 35], a family of computer algorithms aimed at providing information about data without being explicitly designed to deal with that data. A machine-learning algorithm can be exemplified with a spam filter of an email client. This (simple) algorithm presents a (usually large) number of emails that are tagged as ‘spam’ or ‘ham’—often called a ‘training set’—and provides the likelihood that a new inbox email is spam by comparing the wording employed by this latter with the former dataset [36]. The structure based on the relationship between the training set, algorithm and target data is always present in all machine-learning algorithms. More precisely, face-recognition algorithms are based on deep convolutional neural networks, a family of algorithms aimed at finding relevant images (in this case, a face), by subdividing the image into pieces and layers, and subjecting every piece/layer of the image to a machine-learning algorithm (also known as a neural network [37]) until it finds matches with the large number of faces already loaded and tagged in the software (the training set). It is possible to explain in an intuitive way the functioning of the algorithm. As it is known, every image can be represented as a matrix of differently-colored pixel. An image will have three channels, and it is possible to be imagined as a set of three images having, for each pixel, a range from 0 to 255 (three of these numbers form any RGB color). A convolutional neural network approach applies three main steps [29]: the first is the convolution procedure. Convolution aims at preserving the spatial relationship between all pixels by learning some features of the images using relatively small squares of data. A “feature detector” matrix slides on the larger image in order to find particular features of the image (such as edges, curves, or borders) and to produce a series of convoluted images, in which the main features are highlighted. After that, the convoluted images are passed through the “pooling” procedure, namely, a way in which the dimensionality and complexity of the image are drastically reduced: after having defined a spatial neighborhood of a certain *nxn* amount of pixels, the algorithm produces a rectified feature maps, which result by averaging a *nxn* sub-matrix of pixels in a single, average (or maximized) pixel. The rectified feature map is thus smaller and more manageable than the original one. After these two steps (which can be repeated more than one time, according to the complexity of the original image and the aim of the algorithm), the third step is to set up a fully connected layer perceptron (a basic feature of neural networks) that, by means of backpropagation algorithms, compares the rectified (set of) feature map(s) with a training set. At the end of this procedure, the algorithm’s output is a probability distribution telling us whether a certain image contains a face, and the features of that face (age, gender, emotion that the image transmits etc.). The entire procedure is exemplified in Fig 1.

Once the algorithm recognises a face, and if the training set is sufficiently rich with information, new pieces of information on the recognised face can be derived, such as the mood that the face transmits [25], the gender of the face, and a rather refined estimation of the apparent age of the face [25, 26].

**Fig 2:**
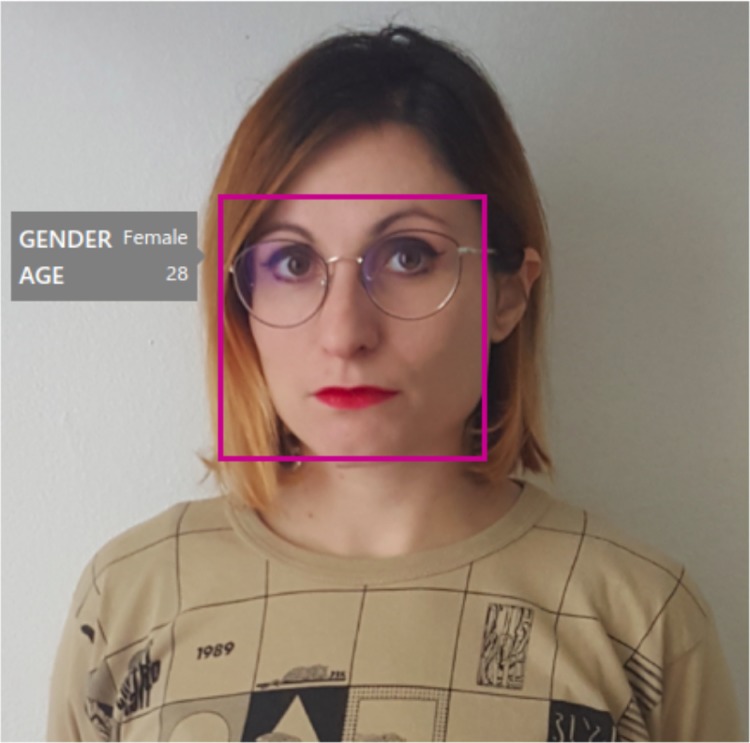
An example of the output of Microsoft Vision CNN algorithm (Printed under a CC BY license, with permission from the subject of the picture, original copyright 2018).

Deep-learning algorithms are computationally demanding and need special computer architectures to be performed efficiently: for instance, employing face-recognition algorithms by means of usual CPUs can be almost computationally unfeasible, even if the number of images to analyse is relatively small. By contrast, Graphic Processing Units (GPUs, namely, the microprocessors of graphic cards) are required to complete medium-difficulty tasks in a reasonable time. More recently, several relevant firms in the IT world have developed Application Programming Interfaces (APIs) that facilitate the transfer of the computational burden, as well as the algorithm complexity, to more equipped remote machines. The analyses presented in this article are based on Microsoft Vision, a commercial cloud API that employs deep convolutional neural networks to identify faces in images and to derive age, gender, expression, facial hair and so on. An example of the working of the API can be seen in Fig 2. The image that the machine-learning algorithm was ‘fed’ with represents a 32-year-old woman. In addition to other pieces of information, the algorithm correctly predicted that the object in the image is a female and misjudges the age by about 4 years (the predicted age is 28).

One question that might arise concerns whether the algorithm can be so accurate in any case, with any image. Previous research [25] has tested the level of precision of these types of algorithm by employing images with known age and gender, concluding that the tolerance level (estimated with a mean square error) is around 5-7 years (the prediction in Fig 2, thus, is quite precise with respect to the normal performance of the algorithm). On the other hand, the capacity to guess the gender of the individual correctly is over 90%. In order to test the precision of the algorithm that we were going to use—the Microsoft Vision algorithm—we applied it to a dataset of pictures for which the age and gender were already known. More specifically, we employed a random subsample of the WIKI dataset [38]. The results largely confirmed previous research, showing that the Microsoft Vision algorithm presents a mean square error for the age prediction of 6.3 years and correctly guessed the gender of the individual represented in the picture in almost 97% of cases (for more information about the analysis, see S1 Fig).

## The case study: The Brexit campaign

To test the validity of the CNN algorithm, we applied this technique to the case of the European Union membership referendum (i.e. Brexit referendum), held in the United Kingdom on 23 June 2016. We selected this case because of its political relevance for the entire EU and the effective confrontation between the two views of ‘out’ or ‘in’: as observed by Seaton [39], this “oversimplicity fits the new architecture of online communications very well”. A second reason is the centrality acquired by online social media during the election campaign, especially in terms of shaping the Brexit debate. Several scholars have tried to clarify the influence of social media for the Leave and Remain sides by observing the social and discourse dynamics behind the referendum [40], by investigating the role of bots on the direction of discussions on Twitter [41], and by mapping the determinants of polarisation and the trends of core narratives on online debating [42].

What is still missing is an understanding of the characteristics of the people involved in these online conversations. We decided to focus on the Brexit case also because it easily allows us to assess the validity of this automatic recognition technique for the social and political sciences, by comparing data derived from Facebook with opinion survey data issued by the BES online panel. Studies based on this dataset showed that older voters with lower levels of education leaned toward Leave, while the ‘winners’ of globalisation—the younger and highly educated professionals—favoured Remain [43]; concerns about immigration and the loss of national identity were important to many who favoured Brexit, and these issues clearly divided the Leave and Remain camps; the British electoral body emerged thus as extremely divided along centre/periphery, social class, education, and age.

## Data

The data in our analysis were extracted by sampling from the Facebook accounts of those individuals who ‘liked’ posts on the official Facebook pages of the Labour party, the Conservative party and UKIP, and their leaders (David Cameron, Boris Johnson, Jeremy Corbyn, and Nigel Farage) between 9 and 22 June 2016 (the period starting and finishing at 00.00GMT). Since the Conservative Party was openly divided in the campaign, with David Cameron as the most prominent Remain campaigner and several cabinet members, including the former mayor of London, Boris Johnson, campaigning to leave the EU, we opted to include Johnson among the leaders whose official page’s data were collected. We selected the three most important parties that gained at least 10% of the votes in the 2015 general election: Conservative Party (36.8%), Labour Party (30.5%) and UK Independence Party (12.7%). The sampling strategy comprised a multistage sampling with two stages: first, a random sample of 13 posts were collected for every page; and second, information was gathered on those people who liked every post. The information was collected in October 2017 using the RFacebook library [44], which in turn, employed the Facebook Graph API [45]. Although recently API access was tightened [46], it is still possible to obtain information that we need from datasets that were collected before February 2018, being the profile picture stored in a fixed URL. A further random selection of users was performed, in order to have a random sample of 1,500 users for every page. After having erased double entries, namely, sampled users who appear more than one time for having liked more than one post, the total sample (originally of 10,500 users) was composed of 9,780 unique users. The profile images of these users were then subjected to the automatic Microsoft Vision face recognition algorithm (Information and descriptive statistics about all the dataset employed in the paper are presented in S1 Table).

As largely expected, not everyone in the sample had uploaded a profile picture that actually represented him/her. More precisely, some of them had not uploaded a picture of a face at all, preferring landscapes, flowers, pets, cars, etc. For every non-recognisable face, the Microsoft Vision algorithm produced a ‘guess’ of what the image represented and categorised it among a set of 88 categories (for instance, when representing a cat, the algorithm returned an *animal*_*cat* string). Although information on the ‘non-human faces’ could be interesting for future research, we decided not to present it in this paper.

The top panel of Table 1 shows that the algorithm was able to recognise a face in about 45% of the cases. In all the other cases, the profile picture represented something that could not be identified as a human face by the CNN algorithm. Given the nature of the data, it is impossible to estimate precisely whether some form of selection is present in the data. It is likely, however, that the sample of detected people did not approximate to a simple random sample of the users who interacted with parties on Facebook. We focus more on this issue in the last section of the paper.

**Table 1:** Table rates of CNN face-detection procedure.

Whole sample	%
Detected faces	44.8
Non-detected faces	55.2
Total	100.0 (n = 9,780)
Subsample	%
Detected but not correct	24.5
Detected and correct	75.5
Total	100.0 (n = 1,000)
Subsample (without under-13 detected)	%
Detected but not correct	19.2
Detected and correct	80.8
Total	100.0 (n = 933)

**Fig 3:**
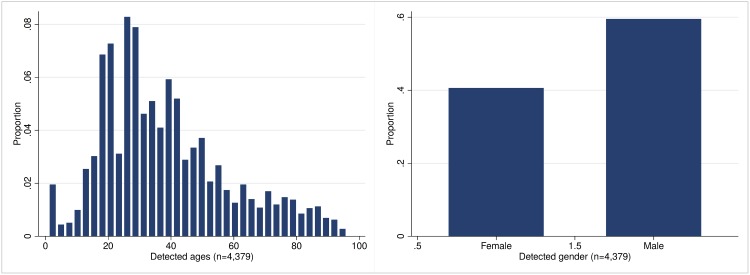
Age and gender distribution detected with the CNN algorithm.

Fig 3 presents the age and gender distributions of the 4,379 people recognised by the algorithm. With regard to gender, as mostly predictable, we see a substantial gender gap: in other words, users who like politicians’ and parties’ posts are more likely to be men than women (60% vis-a-vis 40%). Concerning age, as can be seen in the figure, the mode of the distribution is around the early-30s (32).

One interesting, and potentially problematic, characteristic of the age distribution is represented by its left tail. As it is possible to see, there is a non-irrelevant share of users coded as under-13. This represents a clear bias in our data, since Facebook policy forbids anyone under-13 to have a Facebook account—moreover, genuine profiles of under-13 users are generally banned [47]. A manual assessment of those photos and users—see below—led us to infer that those users had uploaded pictures of their sons and daughters instead of their own face, with the result that the algorithm incorrectly tagged those users as under-13. This issue leads us to think more carefully about the validity of the data produced by the face-recognition algorithm. In the following section, we make a first attempt to estimate how much we can trust the age and gender extracted from the algorithm, by producing a taxonomy of possible biases that can plague our data.

## Validity of the CNN algorithm

### Construct validity of CNN-based age and gender measures

By relying partly on the literature [48] and partly on our analysis of the data, we can enumerate at least four orders of biases that can affect the validity of the CNN algorithm:

1. The profile picture does not represent a human face. This is the case with people who, instead of their own representation in the profile picture, opt to place a landscape, a flower, their pet or whatever is not, strictly speaking, a human face. As indicated in the previous section, this represents just over half of the profile images in our sample (55.2%).
2. The profile picture represents someone else’s face: this is the case where people upload faces of famous characters or, more likely, photos of their children, parents, or their wife/husband. A similar situation arises when there is more than one human face in the picture: which one is the picture that correctly identifies the user?
3. The profile picture is not updated. This issue concerns profile pictures that represent the users, but which are extracted from old photos in which the user is much younger than he/she is now.
4. The profile picture does not transmit to the algorithm the true biological age of the users. In this case, many elements related to the picture itself (the light, image filters, etc.) may intervene in biasing the correct age. Previous literature shows that profile pictures are a crucial tool by which people construct their personal identity online [49]. In some cases, this mechanism could lead users to select pictures that transmit an improved self-representation, leading people to look, for instance, younger than they are. Alternatively, people’s lifestyle (diseases, alcohol or drug abuse, etc.) can transmit an apparent age that is different from the real, biological age, or, more rarely, an image that conveys an incorrect gender.

All in all, it is difficult to find effective solutions for instances related to (1), and the bias of (4) is known, since we already have a measure of algorithm’s precision. However, it is possible to try to assess whether issues (2) and (3) affect our age and gender estimates. In order to do this, we manually investigated profiles of a subsample of 1,000 detected faces. The manual estimation was performed by a single coder, which assessed whether the gender and age of the profile pictures represented a plausible (and updated) guess of the age and gender of the users. The coder relied on the name of the user to assess the gender, and, by inspecting the profile of the user, manually evaluated whether the age would be substantively compatible with the profile image and, whenever possible, with other public pictures present on the user’s page.

The results of this analysis are presented in Table 1 (central and lower panels). As can be seen, the percentage of people who show both a plausibly correct gender and age amounts to more than 75% of our sample. The sample presents a non-residual number of individuals (about 7%) who are estimated as under the age of 13. As expected, these users are fathers and mothers placing a photo of their young children instead of a picture of themselves (none of the detected users of the subsample taken into consideration appeared as a genuine under-13 user). If we erase the 7% of these ‘impossible’ users, the rate of correctly estimated profiles is slightly over 80%. Assuming that the random subsample of 1,000 cases is a realistic representation of what happens in the broader sample (and, more generally, among the British political audience on Facebook), we can derive that about 34% of the age/gender of all 9,780 users sampled are recognised in a sufficiently precise way (keeping constant the 6-year precision bias emphasised above). It is important to state that the CNN-based method of estimating individual characteristics that we adopted here seems to be much more effective than previous methods: users’ age, for instance, is detected for about 30% of our sample, while other methods [20] were able to identify the same characteristic for only 0.3% of a sample of Twitter users. Moreover, although applied here only to Facebook data, we must stress that the method could also be used on Twitter profile pages (Twitter’s API allows to access to profile pages [50]).

**Fig 4:**
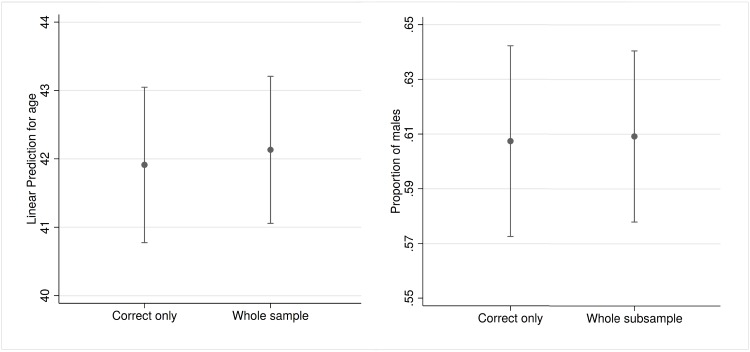
Average age (left panel) and gender proportions (right panel) of correct and the whole subsample and correct-only guess (over-13 detected users only, n = 934 and n = 754).

The 1,000-case subsample also allows to determine of whether the pictures tagged as not correct are systematically different from the correct images. Fig 4 estimates the average age and gender proportion for the entire subsample (933 cases, 93.3% of the estimated over-13 profile pictures. Average age and gender proportions have been calculated by means of linear and logistic models in which the dependent variable is age/gender and the regressor is a dummy variable that identifies correct and incorrect guesses) and the manually derived correct subsample (754 cases, 75.4% of the same subsample). The difference between the whole sample and only plausible ages is small (about 0.2 years of difference) and non-significant. The same can be said for gender. In other words, even if the algorithm does not exactly predict the age and gender of some of our profile pictures, the incorrect guesses do not alter the estimates. The bias, thus, might lead to additional stochastic noise and larger confidence intervals, but it does not significantly affect point estimates.

### External validity of the measures: Comparing CNN with survey data

So far, we have assessed the validity of the measure by employing what could be called a construct-validity approach [51]. In other words, we determined whether the measure that we use—obtained through a CNN algorithm—actually tackles the theoretical concepts of biological age and gender on the users.

In order to make our argument stronger, we tested what might be called the ‘external validity’ of our measure. We did so by comparing the average age and gender proportions collected with the CNN algorithm with those derived from a sample of citizens interviewed during the Brexit election campaign in the context of the British Election Study (BES) research project [52]. The BES online panel is a dataset that, since 2014, follows a sample of people during several election dates. The data cover the European elections of 2014, the national elections of 2015 and the Brexit referendum of 2016. Our argument here is that if age averages and gender proportions are roughly consistent with what emerges from the survey data, we might have additional evidence of the fact that the detected age and gender of users generated by the CNN algorithm is in line with the ‘gold standard’ of data collection methods (namely, a survey).

In order to compare the two datasets, we calculated, for the CCN data, the mean age and gender proportion of those people who liked posts of Pro-Brexit (UKIP, Farage and Johnson) and Anti-Brexit (Labour, Corbyn, Conservatives and Cameron) pages. In the supporting material, the predicted mean age and gender proportions for every party/leader considered are provided (S2 Table and S2 Fig), as well as coefficients of the regression models fitted to calculate the predictions (S3 Table). By operating a quite crude simplification, we defined the first group as the leavers and the second group as the remainers. Also, to allow comparability between the two datasets, we left-trimmed our Facebook dataset on the CNN-predicted age to include only over-18 users (indeed, the BES dataset presents only people over 18 years old). For the BES dataset, we calculated the weighted average age and gender distribution for leavers and remainers, by employing a variable collected in the wave preceding the Brexit campaign (wave 8, collected during June 2016), which measures the voting intention of the survey respondents in the Brexit referendum. Of course, not every individual in the BES sample has a Facebook account or follows politics on Facebook. The calculation of age averages and gender proportions was thus limited to those who declared themselves as having a Facebook account and who followed politicians and parties through this medium (a selection that led to 1,184 cases for the age calculation and 1,285 cases for gender calculation). We must stress that these latter two variables, used to select our reference population (namely, those who follow politics on Facebook), were collected during the 2015 national election campaign wave (more precisely, wave 5 of the panel, collected between March and May 2015). This may lead to a certain amount of additional bias of survey-reported age and gender (namely, the people who followed the national election campaign are not necessarily those who followed the Brexit campaign and vice versa).

**Fig 5:**
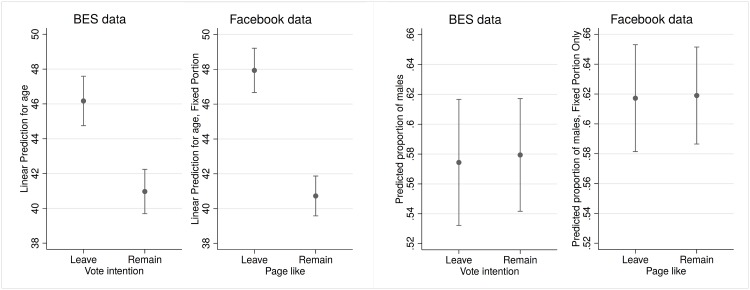
Average age (left panel) and gender proportions (right panel) in BES 2016 data and predicted by CNN model (Facebook data).

Fig 5 shows the mean age and the calculated proportion of men for BES 2016 and for our Facebook data. With regard to the Facebook data, the estimates were obtained by means of a multilevel model (linear in the case of age and logistic in the case of gender), drawing information from leaders/parties’ posts. Survey data means are estimated by employing conventional OLS and logistic regression models and correcting them with survey weights (see S2 Table for the coefficients that produced Fig 5.) As can be seen in the figure, we have added confidence intervals for both the CNN algorithm and the survey data. Of course, the interpretation of the confidence intervals is just indicative of the inferential error and not strictly statistical in method.

The average age and gender proportion of the Remain supporters are pretty similar: the age of those we assumed as ‘remainers’ on Facebook and those who declared their Remain vote in the survey are both around 41, while the detected male remainers are about 58% of the sample vis-à-vis almost 62% of the CNN algorithm. The case of leavers is slightly different: if the difference for gender is the same as for Remain supporters (58% vis-a-vis 62%), the leavers detected on Facebook seem to be slightly older than those in survey (about 48 years old vis-a-vis 46). As stated above, although this is the best that we can manage with the available data, this comparison is not ideal, for several reasons: first of all, we are deducing that those who liked pro-Leave or pro-Remain Facebook posts are going to be, automatically, leavers or remainers; this is a rude simplification, especially if we add that we are only considering those who do one action, namely, liking a certain party/leader post. In addition, the selection of cases to estimate the age and gender of leavers and remainers in the BES dataset presents certain issues, particularly concerning the timing of the collection of the selection variables (a year earlier). However, these problems seem to lead to biases that are negligible (the most important differences being about 2 years for average ages and 4 percentage points for gender).

The CNN-based data show a remarkable consistency with that obtained from survey data: this result reinforces the strength of the evidence from both sources of information. Consistent with the literature about the Brexit referendum, moreover, older people tended to lean towards Leave, and younger towards Remain, while no significant differences were evident in terms of gender [53]. The following section aims at applying CNN-based data to a substantive issue, by investigating which Facebook users answered more promptly to parties’ mobilisation appeals during the Brexit campaign.

## Employing CNN-derived data for the analysis of party digital mobilisation

In the last few years, digital mobilisation, especially through Facebook, has gained increasing relevance in election campaigns [54]. Although the importance of digital communication is clearly established, and there is evidence of the positive relationship linking digital mobilisation with political engagement [55], research has mainly illustrated the characteristics of online mobilised users through surveys or experiments [15, 56, 57]; and data derived from the analysis of social media interaction between politicians and fans are virtually absent. In this section, we employ the face-recognition algorithm shown above by providing new evidence on digital mobilisation. In particular, we consider a rhetorical tool of online political communication as an indicator of mobilisation, one that was very effective in involving voters and activists—the so-called ‘call to action,’ namely, a message in which politicians engage voters by inviting them to share with others (online and offline) an idea conveyed in a post.

### Call to action as a tool of digital mobilisation

Different studies have focused on calls to action in online social media campaigns, showing: (i) that these rhetorical devices are the most used in social media campaigns that eventually emerge as victorious (such as the 2012 Obama campaign [27]); (ii) that, when employed, this kind of message generates high levels of interaction (such as likes, shares, retweets and so on), which makes it easier to diffuse political messages online. Several scholars have argued that this happens because people tend to prefer dialogic and mobilisational messages: by means of such community-building updates, parties and leaders engage in conversation with their people, showing that the party or leader is genuinely attempting to reach both the stable and the potential electorate and, possibly, aiming to assemble an online community of supporters [28, 58]. The literature investigating mobilisation through social media does not explicitly test the effect of these basic characteristics of the audience, and when it uses those characteristics as controls it provides inconsistent results: Vaccari [55], placing the two pieces of information as control variables in an experimental setting, showed that age is not statistically significant, and that women appear slightly more engaged than men. Aldrich and colleagues [56], using survey data, found that online contact appears to be able to mobilise older voters in the US, while indirect online contact is particularly important in mobilising younger voters to become involved in the campaign. Carlisle and Patton [15], using a Facebook users’ survey, found no evidence that age and gender correlated with the political participation of Facebook users during the 2008 US election.

With respect to age, at least two theoretically-founded expectations can be identified. On one hand, an important amount of literature has stated that activism (even in its most inexpensive forms, such as the one taken into account) is a matter of cohorts. In particular, several studies [59, 60] showed quite convincingly that political activism is mainly engaged in by adults, while young people tend to avoid even less expensive forms of political voice. On the other hand, since we are referring to social media, we must take into account that social media participation, mainly by people who are aware of the tool they are dealing with, might be influenced by a cohort-based digital divide of computer skills, which leads older people to be less prone to participate actively in the online community [61–64]. In this way, the sense of community that ‘call to action’ posts aim at fostering in the audience would be disproportionately felt by younger people, lowering the average age of individuals who have interacted with call-to-action posts. The two alternative hypotheses thus read as follows:

*H1a: Older people, more accustomed to political activism, will be more likely to respond to call-to-action posts on politicians’ and parties’ Facebook pages*.

*H1b: Younger people, more accustomed to responding to online social media posts, will be more likely to respond to call-to-action posts on politicians’ and parties’ Facebook pages*.

With regard to gender, on the other hand, we know that men generally exhibit more pronounced activist traits than do women [65], and we do not find any other counter-argument that should lead to an inverted gender gap related to the social media framework. For this reason, the hypothesis concerning gender reads as follows:

*H2: Men, more accustomed to political activism, will be more likely than women to respond to call-to-action posts on politicians’ and parties’ Facebook pages*.

### Data and models

The data are drawn from the dataset shown above and include the CNN-algorithm-based variables. In this case, only the detected profiles of people who are identified as over 13 years old were taken into account. In total, 4,153 profiles had responded to 56 posts of leaders and parties for both leavers and remainers during the two weeks preceding the election. In order to define the messages containing a call to action, the posts were content-analysed by a coder, who was blinded to the identity of the sender of each message. The posts were coded as 0 in the absence of call-to-action messages, and 1 in instances where such a message was detected (e.g. messages containing “SHARE to let friends know” or “Can you help? SIGN UP now!” were defined as call-to-action messages). About 25% of the messages were coded as call-to-action messages. These messages were mainly published by the pro-Brexit parties and leaders (34% vis-a-vis 17%). This is consistent with the results of Auter and Fine [54], who found that challengers and not-established movements especially employ online mobilisation. In the models that follow, we use the CNN-detected age and gender of the Facebook users as the dependent variable and the ‘call to action’ nature of the message as the independent variable. Although it can seem peculiar employing a model in which the age and gender are dependent variables, we have to keep in mind our aim of assessing whether a characteristic of politicians’ and parties’ posts aimed at involving different people. In this case, the detected age/gender of users was a consequence of a certain political message. In addition to our main dependent and independent variables, we added some confounders, which can alter the relationship of interest: the pro- or anti-Brexit nature of the page; the number of likes, comments and shares of each post; the distance of the post from the election day; and the hour in which the post was published (from 8.00 to 12.59, from 13.00 to 18.59, and from 19.00 to 23.59). The models we will employed are multilevel random intercept models (with linear and logistic specifications according to the nature of the dependent variable), in which users are nested into posts.

### Results

The results of the models for detected age and gender are presented in Table 2. For the control variables, as the number of likes rises, the predicted age lowers (while there is no difference for the detected gender). By contrast, a higher number of comments leads to a (marginally) higher average age and proportion of males. The number of shares leads to a marginal change in the propensity for women to like the post. The distance from the election day does not lead to any change in the dependent variable, while the hour of publication indicates that the male proportion is lower in the afternoon. A post published by a pro-Brexit page leads to higher average age (and the variable makes no difference to the detected gender). This is consistent with studies that showed that pro-Brexit people tend to be older than remainers. Also in this case, pro- or anti-Brexit sources do not alter the gender gap. As can be seen in Model 1, the coefficient of call-to-action messages is positive, meaning that people who react more promptly to calls to action are older than individuals who respond to posts in which no call is present. Although quite small, a difference of two years on average (42.5 vis-a-vis 44.7), the effect is significant to the 5%. Model 2, on the other hand, shows that there is no gender gap between those who react to call-to-action messages and those who respond to those messages.

**Table 2:** Detected age, gender and nature of the message (call to action vs. any action).

Independent variables	Detected age		Detected gender	
	Coef.	S.E.	Coef.	S.E.
Call to action (ref. Any call)	2.19**	(0.96)	0.11	(0.12)
Remain supporter page (ref. Leave)	-8.04***	(0.80)	-0.02	(0.10)
Number of likes (/100)	-0.02*	(0.01)	-0.00	(0.00)
Number of comments (/100)	0.04*	(0.02)	0.01***	(0.00)
Number of shares (/100)	-0.03	(0.03)	-0.01***	(0.00)
Distance from the election day	0.08	(0.10)	0.02	(0.01)
Hour of post publication (ref. 6.00/12.59)				
13.00/18.59	-0.31	(0.77)	-0.23**	(0.10)
19.00/23.59	-2.66	(1.66)	-0.21	(0.21)
Constant	48.78***	(1.35)	0.60***	(0.18)
Level-2 variance (ln)	0.26	(0.34)	-1.67***	(0.23)
Level-1 variance (ln)	2.84***	(0.01)		
Observations	4.153		4.153	
Number of groups	56		56	

Standard errors in parentheses

*** *p* < 0.01,

** *p* < 0.05,

* *p* < 0.1

The application of the CNN algorithm to the study of digital mobilisation made it possible to clarify the basic characteristics of the users who interacted online with Brexit, pointing out that, on the one hand, older people are more likely to answer call-to-action messages, corroborating hypothesis H1a. On the other hand, the gender gap hypothesis was not corroborated by our data.

## Discussion, caveats and future research

In this paper, a novel method for identifying basic characteristics (age and gender) of social media users has been presented. The method employs a convolutional neural network algorithm to determine apparent age and gender of Facebook users’ profile pictures. To our knowledge, this is the first attempt to employ such an algorithm to gather information about social media users in order to study their political behavior. By employing Facebook data, we collected a sample of users who ‘liked’ a certain political party/leader’s post during the two weeks before the Brexit referendum. We showed that the algorithm is able to identify human faces and detect their age and gender in about 45% of cases. After having manually investigated the results of the algorithm on a subsample of the data, we estimated that about 75% of the detected profile pictures were correct (by ‘correct’, we mean that the age and gender of the user were apparently consistent with what the user presented in his/her profile picture and other pictures within the profile). In order to strengthen our argument, we verified the consistency of CNN-derived information with data gathered through traditional surveys. This latter implies that these two techniques reinforce each other: on the one hand, CNN data is corroborated by well-established sources of data; on the other hand, surveys have a confirmation of their validity derived directly from the users of the digital environment analysed. Finally, we applied our measures to digital mobilisation that had occurred during the election campaign and especially to the call-to-action messages published by parties and leaders on Facebook. Data from the CNN algorithm allowed us to identify that users who responded more promptly to mobilising messages were slightly older, while no difference was reported in the gender gap.

From the methodological perspective, a first element of interest in this new technique emerges when comparing it to other methods employed in the literature. To obtain data ‘in nature’—namely, not by relying on directly surveying the social media audience—we can basically count on two methods: the first allows the derivation of psychological and demographic characteristics of users by analysing the vocabulary and employment of certain words; and the second relies on meta-data directly provided by the user in order to derive variables such as age, gender, social class, etc. Whereas the former approach is extremely demanding in terms of single users’ data availability, the latter one provides information for only a small portion of the universe of the Twitter sphere [19, 22]; moreover, this method is not employable on other social networks, such as Facebook or Instagram, in which that kind of information are not derivable. Our CNN technique has been tested on 2016 data collected on Facebook, a social network for which no meta-data-based techniques were available (however, nothing would prevent us from applying the same algorithm on Twitter, given the ease with which it is possible to obtain users’ profile pictures [50]). The real advantage of the CNN approach, however, emerges when we compare the efficiency of the methods: if meta-data techniques can correctly detect only small quotas of users (Sloan and colleagues [20] can detect the age of about 0.3% of their data), the proposed technique, by guessing about 30% of the sample, seems much more promising in inferring this basic information.

The proposed algorithm also presents some drawbacks. As with other meta-data-based techniques, the face-recognition algorithm is only able to guess a certain quota of our universe (with the consequence that we have no information about the non-detected users). As a result, our analysis is based on a non-random sample of the population, namely, we have no evidence to assess whether the group of people who were not detected (those who, for instance, place the photo of their cat in the profile picture) present the same characteristics as those who we have been able to guess. The second limit of the paper resides in the algorithm employed to detect that information: the technique gives us a machine-learning-based guess of the apparent age and gender of an individual, but we have no information declared by the user him/herself. Although we have estimated that about 30% of the sample can be plausibly guessed, we do not have the absolute certainty that the person portrayed is an actual representation of the user and/or that the age and gender of the person are a faithful prediction of their real characteristics. The third, and final, limit of the paper is that the technique proposed here is based on a proprietary algorithm, developed by a private company, the in-depth the technical details of which we are unable to discover. This limit, as stated above, does not reside in the availability of the algorithm itself. Face- and image-recognition algorithms are widespread, and many of them are open source/free software [66, 67]. More pragmatically, it is hard to run this software on consumer machines because of its voracity of computational power. We thus decided to propose a method that could be employed by social scientists without being forced to rely on expensive machines and complicated machine-learning algorithms.

Beyond the potential drawbacks of the method, however, the use of data from the CNN algorithm has shown that its application can lead to new evidence and can be fruitfully used in social and political science studies. In particular, a non-exhaustive list of further developments of CNN applications can lead to: (i) confirmation of the validity of survey data and obtaining information from those populations that are generally unavailable within traditional surveys; (ii) assessment of whether older/younger people tend to support older/younger leaders; (iii) determination—by collecting more data—of how the population of politician/parties change according to their political strategies; (iv) assessment of whether the gender gap is still present in parties that are more concerned with gender equity issues by employing comparative data (relatively easy to collect on social media); (v) inference of users’ localisation from their surname; and (vi) inference of socio-economic information through the analysis of users’ posts. We are confident that the growing importance of digital environments and, in particular, of social media interactions requires new tools to integrate current techniques of the investigation of political and social reality—mainly based on opinion surveys—with data coming directly from users. We are persuaded that the CNN algorithm, as well as machine-learning approaches more generally, will become increasingly useful to scholars in answering a large number of substantive questions.

## Supporting information

S1 Fig Distribution of differences between real and predicted age (from a subsample of the WIKI dataset, n = 1,000). Although previous research [25] has tested the level of precision of CNN algorithms by employing images with known age and gender, concluding that the tolerance levels (estimated with a mean square error) are around 5-7 years for age and over 90% for gender, we decided to test the performance of the CNN algorithm that we use (Microsoft Vision). To test the CNN algorithm’s precision, we employed the WIKI dataset [38] of 61,867 pictures, collected from Wikipedia, of which the age and gender were already known. We sampled 1,000 pictures and we subjected them to the CNN algorithm. In the results, the gender predicted by the algorithm was incorrect in only 34 cases out of 1,000—a precision level of almost 97%. With regard to age, the mean square error of the difference between real and predicted ages (namely, the square-root of the squared differences, divided by the number of cases) is 6.3 years. Picture A1 shows the distribution of the differences between real and predicted ages. The distribution is not very skewed (skewness = -0.09), meaning that the algorithm does not tend systematically to over- or underestimate ages in pictures.
(EPS)

S2 Fig Average age (left panel) and gender proportions (right panel) by party/leader considered. (EPS)

S1 Table Descriptive statistics of the datasets employed. (PDF)

S2 Table Models’ estimates of S2 Fig. (PDF)

S3 Table Models’ estimates of Fig 5. (PDF)

S1 Dataset Datasets employed in the article. (ZIP)
