# Supplementary material for: Using deep-learning algorithms to derive basic characteristics of social media users: The Brexit campaign as a case study
Source: PLoS One. 2019 Jan 25;14(1):e0211013. doi: 10.1371/journal.pone.0211013 (PMC6347201; doi:10.1371/journal.pone.0211013)
Supplement: S1 Table — (PDF) [file pone.0211013.s003.pdf]

S1 Table. Descriptive Statistics of the Datasets Employed.

	Mean/P.	S.D.	Min	Max	N
	Detected Facebook users				
Age (predicted)	.59	.49	0	1	4379
Gender: Male (ref. Female)	41.0	19.2	0	95	4379
Remain fans (ref. Leave fans)	.57	.49	0	1	9780
Farage fans	.14	.35	0	1	4379
Corbyn	.15	.35	0	1	4379
Cameron	.15	.36	0	1	4379
Johnson	.13	.34	0	1	4379
UKIP	.12	.33	0	1	4379
Tories	.16	.36	0	1	4379
Labour	.14	.35	0	1	4379
	Facebook posts				
Likes count	4522.6	4799.4	58	20516	56
Shares count	1342.7	1974.7	0	10241	56
Comments count	879.9	1910.2	13	14271	56
Distance from the election day	6.5	4.0	0	13	56
Hour: 6.00/12.59	.43	.49	0	1	56
Hour 13.00/18.59	.52	.50	0	1	56
Hour: 19.00/23.59	.05	.23	0	1	56
Call to action	.30	.46	0	1	56
	WIKI subsample				
Age (actual)	38.2	16.4	13	95	1000
Age (predicted)	39.7	14.3	9	93	1000
Gender (actual)	.74	.44	0	1	1000
Gender (predicted)	.76	.43	0	1	1000
	BES sample				
Age	48.8	16.4	15	82	1285
Gender	0.57	.50	0	1	1285
Remain vote intention (ref. Leave)	.58	.49	0	1	1285
