# Supplementary material for: Using deep-learning algorithms to derive basic characteristics of social media users: The Brexit campaign as a case study
Source: PLoS One. 2019 Jan 25;14(1):e0211013. doi: 10.1371/journal.pone.0211013 (PMC6347201; doi:10.1371/journal.pone.0211013)
Supplement: S2 Table — (PDF) [file pone.0211013.s004.pdf]

S2 Table. Models' Estimates of S2 Fig.

Indep. variables	Dep. var. age Linear ML model) Coef.	S.E.	Dep. var. gender (Logistic ML model) Coef.	S.E.
Party/Leader (ref. Farage)				
Corbyn	-6.88***	(1.18)	-0.27*	(0.14)
Cameron	-8.93***	(1.15)	0.49***	(0.14)
Johnson	-0.02	(1.17)	0.15	(0.14)
UKIP	3.96***	(1.22)	0.19	(0.15)
Tories	-4.84***	(1.22)	-0.01	(0.15)
Labour	-4.29***	(1.18)	0.14	(0.15)
Constant	43.68***	(0.92)	0.35***	(0.10)
Level-2 variance (ln)	0.53	(0.40)	-2.02***	(0.41)
Level-1 variance (ln)	2.83***	(0.01)		
Observations	4.153		4.153	
Number of level-2 units (posts)	56		56	

Standard errors in parentheses
*** $p < 0.01$, ** $p < 0.05$, * $p < 0.1$
