# Supplementary material for: Using deep-learning algorithms to derive basic characteristics of social media users: The Brexit campaign as a case study
Source: PLoS One. 2019 Jan 25;14(1):e0211013. doi: 10.1371/journal.pone.0211013 (PMC6347201; doi:10.1371/journal.pone.0211013)
Supplement: S3 Table — (PDF) [file pone.0211013.s005.pdf]

S3 Table. Models' Estimates of Fig 5.

	Age detected	Age (BES 2016)	Gender detected	Gender (BES 2016)
Indep. variables	Coef.	Coef.	Coef.	Coef.
Remain vote choice/Like	-7.20***	-5.20***	0.01	0.06
	(0.87)	(0.97)	(0.10)	(0.10)
Constant	47.94***	46.17***	0.47***	0.49***
	(0.65)	(0.72)	(0.08)	(0.08)
Level-2 variance (ln)	0.79***		-0.61***	
	(0.19)		(0.12)	
Level-1 variance (ln)	2.83***			
	(0.01)			
Observations	4.11	1.184	4.11	1.285
Number of groups	56	-	56	-

Standard errors in parentheses
*** $p < 0.01$, ** $p < 0.05$, * $p < 0.1$
