# Supplementary material for: Using deep-learning algorithms to derive basic characteristics of social media users: The Brexit campaign as a case study
Source: PLoS One. 2019 Jan 25;14(1):e0211013. doi: 10.1371/journal.pone.0211013 (PMC6347201; doi:10.1371/journal.pone.0211013)
Supplement: S1 Dataset — (ZIP) [file pone.0211013.s006.zip › Datasets.doc]

**Dataset structure**

DataBes.dta is a Stata data file containing a subset of the the British Election Study variables employed in the paper.

dataFb.dta is the dataset of the sampled users, containing predicted age (variable age_int) and gender (gender).

DataFbSubsampleVal.dta is the random sub-sample of dataFb, on which we have derived manually gender and age of our sampled users.

DataPrecision is the wiki dataset plus the prediction of the Microsoft vision algorithm. It is used to assess the precision of the algorithm
